# Intractable Epilepsy in Maternally Inherited Leigh Syndrome (MILS) Due to the Sporadic Variant m.8993T>G in MT-ATP6: A Case Report

**DOI:** 10.7759/cureus.22716

**Published:** 2022-02-28

**Authors:** Josef Finsterer

**Affiliations:** 1 Neurology, Krankenanstalt Rudolfstiftung, Vienna, AUT

**Keywords:** genetics, respiratory chain, mitochondrial disorder, mtdna, leigh syndrome

## Abstract

Epilepsy is a common phenotypic feature of Leigh syndrome. However, data about the characteristics, treatments, and outcomes of epilepsy in maternally inherited Leigh syndrome (MILS) are limited. A four-year-old boy with sporadic MILS developed epilepsy at the age of five months. His condition was diagnosed at birth and was caused by the m.8993T>G variant with a heteroplasmy rate of 90% in skin fibroblasts. The patient presented with absences, and focal and generalized tonic-clonic seizures. Despite treatment with anti-seizure drugs such as vigabatrin, lamotrigine, clonazepam, diazepam, clobazam, and phenobarbital, seizure control was insufficient, and seizures became intractable. The patient died after recurrent aspiration, pneumonia, and sepsis. In conclusion, the m.8993T>G variant can occur sporadically, and m.8993T>G carriers with MILS may develop epilepsy as the disease progresses. Moreover, the treatment for epilepsy in MILS can be challenging, and epilepsy can become intractable and can contribute to fatal outcomes.

## Introduction

Maternally inherited Leigh syndrome (MILS) is a rare, syndromic mitochondrial disorder clinically characterized by early childhood manifestations such as leukoencephalopathy with cognitive impairment, weakness, muscle hypotonia, nutritional disorder, epilepsy, and respiratory failure with a fatal outcome [1]. Genotypically, MILS is caused by mutations in the mitochondrial ATPase6 (MT-ATP6) gene located in the mitochondrial DNA (mtDNA) [2-4]. The most common of the MT-ATP6 variants responsible for MILS is the the variant m.8993T>G [5]. The m.8993T>G variant results in impaired oxidative phosphorylation (OXPHOS) with decreased spare respiratory capacity in response to energy stress [6]. In fibroblasts of m.8993T>G carriers, reduced glycolysis with reduced glycolytic capacity, and reduced ability to switch to glycolysis upon complete inhibition of OXPHOS activities were observed [6]. This dysregulated energy reprogramming leads to a faulty interaction between OXPHOS and glycolysis during an energy crisis [6]. Epilepsy is a common phenotypic feature of MILS occurring in about one-third of the patients, but electroencephalography (EEG) abnormalities can be recorded in three-quarters of cases [5]. However, data about the characteristics, treatments, and outcomes of epilepsy in MILS are limited.

## Case presentation

**Figure 1:**
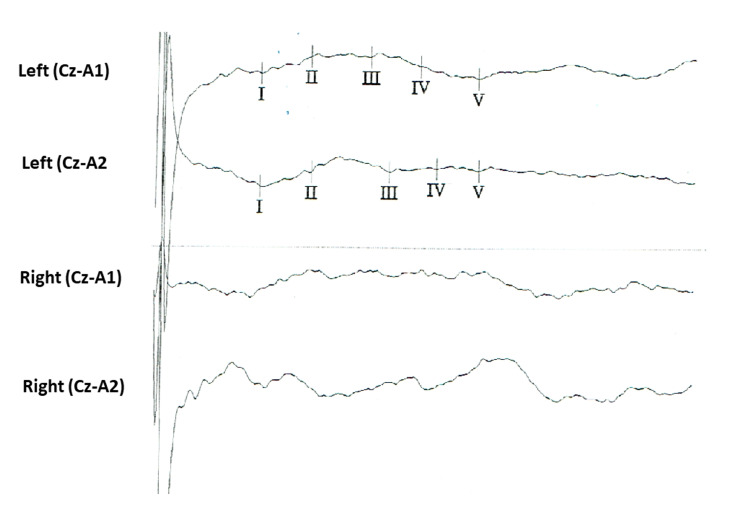
Acoustically evoked potentials showing nonreproducible recordings bilaterally

The patient was a four-year-old boy with MILS. He was born full term after an uncomplicated pregnancy, with Apgar scores of 7, 8, and 9, respectively. Moreover, he required incubator care within the first few hours after delivery. After a few days, the mother noticed that the baby had a weak sucking reflex. Despite her efforts, this led to nutritional disorder. At three months of age, microcephaly, head flexor weakness, and muscle hypotonia were observed. Since there were high lactate levels in the serum and the cerebrospinal fluid, mitochondrial disorder was suspected. Screening for mutations in the mtDNA-localized genes in blood lymphocytes and skin fibroblasts revealed m.8993T>G mutation in MT-ATP6. The heteroplasmy rates of the mutation were 75% in the buccal mucosa, 86% in blood lymphocytes, and 90% in skin fibroblasts. The mother was clinically unaffected and tested negative for this particular mtDNA variant. Cerebral magnetic resonance imaging was considered. However, it was not performed as the patient should be under general anesthesia. Acoustically evoked potentials were not bilaterally reproducible (Figure 1), suggesting impaired hearing. Antioxidants had no significant effects.

**Figure 2:**
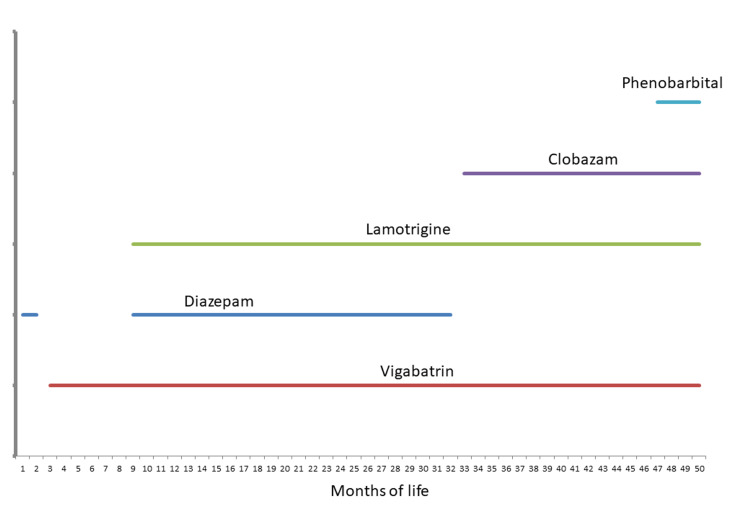
Anti-seizure drugs given to the index patient since onset of epilepsy at age five months

Five months after delivery, the patient developed recurrent absences associated with ocular palsy, each lasting 30-60 seconds. Pediatricians suspected seizures, which were confirmed on EEG. The seizure frequency was high with multiple ictal events per day. Diazepam was administered rectally for each attack. However, it was replaced by vigabatrin at six months of age because of fatigue. Clonazepam was added to the treatment regimen because of insufficient seizure control. Due to continuous inadequate seizure control and fatigue, clonazepam was changed to diazepam at the age of one year. Owing to persistent inadequate seizure control, lamotrigine was included at one year of age, which temporarily reduced seizure frequency. During the absence of seizures, the patient was able to turn his head, speak a few words, eat mushy food, and move his limbs. However, he could not sit or stand without help and remained incontinent. The patient was diagnosed with retinitis pigmentosa at the age of two years. However, his vision was not affected. Due to increasing seizure frequency, clobazam was added to the three anti-seizure drugs (ASDs) at three years of age (Figure 2). Levetiracetam was tried but had to be discontinued because of side effects. Since the treatment was insufficient, phenobarbital was prescribed at four years of age (Figure 2), and it was used for extremely severe seizures. Subsequently, swallowing became an increasing problem. At the age of four years, the boy experienced aspiration, followed by pneumonia complicated by sepsis. Eventually, he died despite maximum treatment.

## Discussion

Interestingly, the current case reports MILS characterized by progressive, generalized, and ultimately intractable epileptic seizure activity. The intractable seizures led to aspiration, pneumonia, sepsis, and eventually death.

MILS is a mitochondrial syndrome that is phenotypically similar to Leigh syndrome and is characterized by early onset (<24 months) and maternal inheritance [7]. Among the pathogenic mtDNA variants responsible for MILS, variants in the MT-ATP6 gene are the most common [7]. The heteroplasmy rates of MT-ATP6 variants in patients with MILS commonly exceed 90% [7]. However, the rates significantly vary between tissues and contribute to intrafamilial phenotypic heterogeneity. In few cases, the condition started >60 months [7].

Compared with other MT-ATP6 variants, the m.8993T>G variant is associated with earlier-onset MILS [7]. Patients with the m.8993T>G variant have a faster disease progression than those with other MT-ATP6 variants [7]. The m.8993T>C variant typically has a milder phenotype than the m.8993T>G variant. MT-ATP6 variants are usually inherited. However, sporadic MT-ATP6 mutations, as in the index case, are rarely reported. The m.8993T>G variant induces a structural defect in the F(1)F(0)-ATPase, which leads to insufficient coupling between proton transport and adenosine triphosphate synthesis with a strong reduction in adenosine triphosphate production.

The phenotypic spectrum of MT-ATP6 variants is broad, ranging from asymptomatic mutation to neuropathy, ataxia, retinitis pigmentosa syndrome, and MILS. Patients with MILS present with psychomotor and mental retardation, failure to thrive, spastic quadriparesis, muscular hypotonia, and lactic acidosis [1]. The other characteristics include hypoacusis, dystonia, chorea, myoclonus, hypertrophic cardiomyopathy, hyporeflexia, and hepatopathy [1]. More than half of the m.8993T>G carriers exhibit hypocitrullinemia. Cerebral imaging shows abnormalities of the basal ganglia or brainstem [7]. Frontal lobe involvement and cerebellar atrophy have occasionally been reported [8]. Sudden apnea is an unusual phenotypic trait of m.8993T>G carriers. About one-third of patients with MT-ATP6 variants present with epileptic seizures. However, three-quarters of patients have EEG abnormalities. Focal and generalized seizures and infantile spasms with hypsarrhythmias are observed [9]. The severity of the disease, prevalence of epileptic seizures, and severity of EEG changes increase with elevated heteroplasmy rates [6].

Treatment with ASDs for epilepsy in patients with the m.8993T>G variant is not consistent. Different ASDs have been applied. Infantile spasms may respond to steroids and vigabatrin [9]. Limited experience is available with ASDs such as perampanel, zonisamide, felbamate, ethosuximide, and topiramate. Gene therapy is effective, at least in cell cultures [10]. However, whether it has an antiepileptic effect is unknown. Since some ASDs have a mitochondrial toxic potential, mitochondrial toxic ASDs should be used with caution among patients with MILS.

## Conclusions

The index case shows that the m.8993T>G variant can be sporadic, that m.8993T>G carriers with MILS may develop epilepsy as the disease progresses, that ASD treatment is challenging in these patients, and that epilepsy can contribute to fatal outcomes. This case is important as only few MILS patients with intractable epilepsy and only a few MILS patients with hypoacusis have been reported. Future research should focus on the genetic treatment of MILS and on the treatment of epilepsy with newly developed ASDs.
